# Post-operative left atrial volume index is a predictor of the occurrence of permanent atrial fibrillation after mitral valve surgery in patients who undergo mitral valve surgery

**DOI:** 10.1186/s12947-018-0123-1

**Published:** 2018-03-09

**Authors:** Min-Kyung Kang, Boyoung Joung, Chi Young Shim, In Jeong Cho, Woo-In Yang, Jeonggeun Moon, Yangsoo Jang, Namsik Chung, Byung-Chul Chang, Jong-Won Ha

**Affiliations:** 1 Division of Cardiology, Kangnam Sacred Heart Hospital, Hallym University Medical Center, Seoul, South Korea; 2 Division of Cardiology and Cardiovascular Surgery, Severance Cardiovascular Hospital, Yonsei University College of Medicine, 134 Shinchon-dong, Seodaemun-gu, Seoul, 120-752 Republic of Korea; 3 Division of Cardiology, CHA Bundang Medical Center, CHA University, Seongnam, South Korea; 4 Division of Cardiology and Cardiovascular Surgery, Department of Internal Medicine, Gachon University of Medicine and Science, Incheon, South Korea

**Keywords:** Atrial fibrillation, Mitral valve, Left atrium

## Abstract

**Background:**

Atrial fibrillation (AF) can occur even after the correction of mitral valve (MV) pathology in patients who have pre-operative sinus rhythm and undergo MV surgery. However, the factors associated with the occurrence of AF after MV surgery are still unclear. The aim of this retrospective study was to investigate the factors determining the occurrence of permanent AF after MV surgery in patients with preoperative sinus rhythm who underwent MV surgery.

**Methods:**

Four hundred and forty-two patients (mean age 46 ± 12, 190 men) who underwent MV surgery and sinus rhythm were investigated retrospectively. Transthoracic echocardiography was performed before and after MV surgery at the time of dismissal.

**Results:**

Permanent post-operative AF occurred in 81 (18%) patients even after successful MV surgery and preoperative sinus rhythm. It was more common in rheumatic etiology, a presence of mitral stenosis, lower pre- and post-operative left ventricular ejection fraction, higher post-operative mean diastolic pressure gradient across mitral prosthesis, larger post-operative left atrial volume index (LAVI) and lesser degrees of reduction in LAVI after surgery. In multiple regression analysis, post-operative LAVI was found to be an independent predictor for occurrence of AF. Post-operative LAVI > 39 ml/m2 was the cut-off value for best prediction of new onset permanent AF (sensitivity: 79%, AUC: 0.762, SE: 0.051, *p* < 0.001).

**Conclusion:**

New-onset permanent post-operative AF is not uncommon, even after successful MV surgery despite pre-operative sinus rhythm. Larger post-operative LAVI was an independent predictor for the occurrence of AF.

## Background

Increased left atrial (LA) size is associated with the occurrence of atrial fibrillation (AF) [1]. Therefore, AF is frequently observed in patients with chronic mitral valve (MV) disease, which invariably induces LA remodeling [2–4]. On the other hand, reduction of LA size (reverse LA remodeling) can also occur after correction of MV pathology [3, 5]. It has been shown that the degree of reverse LA remodeling varies, particularly according to pre-operative cardiac rhythm. Moreover, pre-operative sinus rhythm is associated with larger degrees of reverse LA remodeling [3]. In addition, this structural reversal can also induce reversal of electrophysiologic abnormalities that are predisposed to the occurrence of AF. Therefore, reversal of these changes by treatment, i.e., MV surgery, could potentially have important implications for the prevention of AF [5, 6]. Nevertheless AF can occur even after correction of MV pathology in patients who have undergone MV surgery [7–17]. However, the incidence and predictors of new onset permanent AF after MV surgery have not been clearly defined. Therefore, the aim of this study is to investigate the prevalence and predictors of the occurrence of new onset permanent AF in patients with MV diseases who have undergone MV surgery and sinus rhythm pre-operatively.

## Methods

### Study design and participants

**Fig. 1:**
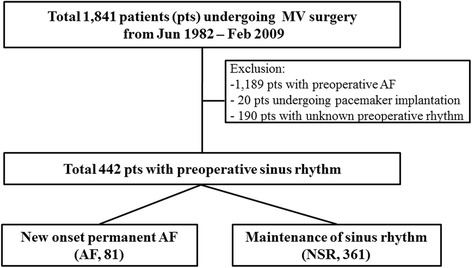
Enrolled study subjects. Patients underwent surgery were sorted to the two groups - new onset permanent AF (AF, 81) vs. maintenance of sinus rhythm (NSR, 361)

A total of 1841 patients underwent MV surgery from June 1982 to February 2009. Among them, patients with pre-existing AF, concomitant MAZE procedure during surgery (*n* = 1189), patients with permanent pacemaker implantation (*n* = 20), and patients unknown pre-operative rhythm (*n* = 190) were excluded. The remaining 442 patients (mean age 46 ± 12, 191 men) comprised the study population (Fig. 1). We reviewed the medical records to define the etiology of MV pathology and reason for valve surgery in addition to pre- and post-operative transthoracic echocardiography (TTE) and electrocardiogram (ECG). ECG was taken pre- and post-operatively and at follow up once a year thereafter. New onset permanent AF was defined as the occurrence of AF post-operatively detected by post-operative ECG during the hospital stay and that persisted thereafter.

### Echocardiography

TTE was performed by standard techniques with a 2.5-MHz transducer. Two-dimensional echocardiographic images were obtained in the standard parasternal long-axis and apical 2 and 4 chamber views. Left ventricular (LV) ejection fraction (EF) was assessed by the modified Quinones method. Mediolateral (ML) and superior/inferior (SI) dimensions of LA were measured from the apical 4 chamber view, and anteroposterior (AP) dimensions were measured from the parasternal long axis view at the end-systole. Maximal LA volume was calculated using the prolate ellipsoid model3 and indexed to the body surface area (LA volume index; LAVI). Measurement of LA volume was available in 200 of 442 patients at pre- and post-operative periods. In those patients, the LA volume change and the percentage of LA volume change were calculated [18]. Assessment of valvuar heart diseases were based on the guidelines [19].

### Statistical analysis

Continuous variables are presented as means ± standard deviation (SD) and compared using Student’s unpaired t test or Mann-Whitney’s U test. Categorical variables are presented as numbers or percentages, and used the Chi-square test. To determine the variables associated with the occurrence of AF, logistic regression analysis was performed separately using clinical variables and echocardiographic findings. The predictive ability of the LAVI was determined by the area under the receiver-operating characteristic curves (ROC). Kaplan-Meier estimator was used for AF free survival curves. *P* value < 0.05 was considered statistically significant.

## Results

**Fig. 2:**
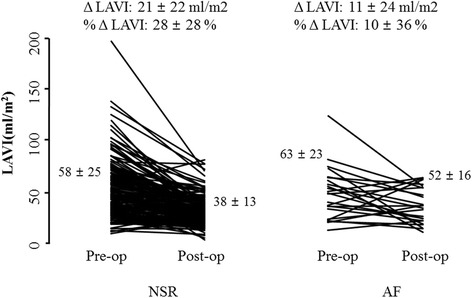
Degree of left atrial reverse remodeling . LAVI, left atrial volume index; Δ LAVI, change of LAVI; % Δ LAVI, percent change of LAVI, NSR, maintenance of normal sinus rhythm; AF, new onset permanent AF

**Fig. 3:**
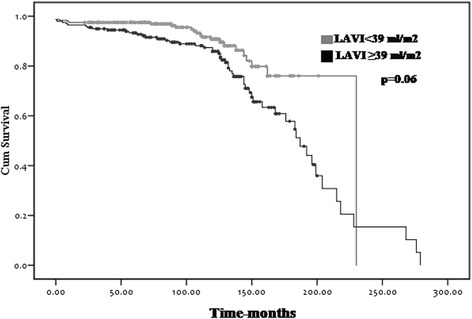
AF free survival curves

The enrolled patients were classified into the two groups according to the occurrence of permanent AF or maintained sinus rhythm after MV surgery (normal sinus rhythm [NSR] group vs. AF group). Post-operative new onset permanent AF occurred in 81 (18%) patients. Baseline characteristics of the study subjects are shown in Table 1. The mean age at the time of surgery was similar in both groups. The majority of patients (81%) had rheumatic etiology and others (19%) had non-rheumatic etiology, such as infective endocarditis, MV prolapse, or chordae rupture. The proportion of rheumatic valve disease was significantly higher in patients with new onset permanent AF (*p* = 0.018). The median interval from the surgery to the occurrence of AF was 9.2 years (110.6 ± 78.9 months), and the mean follow-up duration was not significantly different in both groups (9.8 ± 5.9 years in the NSR vs. 9.4 ± 6.5 years in the AF, *p* = 0.566). The TTE parameters before and after surgery are listed in Table 2. Pre- and post-operative LV EF were significantly lower in the AF group (63 ± 10% in the NSR vs. 59 ± 14% in the AF, *p* = 0.026 & 61 ± 10% in the NSR vs. 57 ± 14% in the AF, *p* = 0.006), although the LV EF of both groups were in normal range. Pre-operative LA AP dimension (51 ± 9 mm in the NSR vs. 53 ± 7 mm in the AF, *p* = 0.080) and LAVI (58 ± 25 ml/m^2^ in the NSR vs. 63 ± 23 ml/m^2^ in the AF, *p* = 0.399) were not significantly different in both groups. However, the degree of reduction of LAVI (21 ± 21 ml/m^2^ in the NSR vs. 11 ± 23 ml/m^2^ in the AF, *p* = 0.041) and percentage reduction of LAVI (28 ± 28% in the NSR vs. 10 ± 37% in the AF, *p* < .001) were significantly smaller in the AF group (Fig. 2). Therefore, post-operative LA size was significantly larger in the AF group, shown as AP dimension (43 ± 6 mm in the NSR vs. 51 ± 8 mm in the AF, *p* < .001) and LAVI (38 ± 13 ml/m^2^ in the NSR vs. 52 ± 16 ml/m^2^ in the AF, *p* < .001). When the degree of reduction of LA dimension was compared according to the direction (AP, ML or SI), the change in SI direction was most prominent (Table 2). Regarding the hemodynamic variables, the post-operative mean diastolic pressure gradient (MDPG) of the MV was significantly higher in patients with permanent AF group (3.5 ± 1.4 mmHg in the NSR vs. 3.9 ± 1.5 mmHg in the AF, *p* = 0.023). The grade of pre and post-operative tricuspid regurgitation (TR) were slightly higher in the AF group. Post-operative estimated pulmonary artery pressure (PAP) was also slightly higher in the AF group. In univariate analysis, rheumatic etiology (odds ratio [OR] = 2.474, 95% confidence interval [CI] = 1.143–5.355, *p* = 0.021), lower pre (OR = 0.971, 95% CI = 0.945–0.997, *p* = 0.028) and post-operative LV EF (OR = 0.970, 95% CI = 0.949–0.992, *p* = 0.007), higher post-operative MDPG across mitral prosthesis (OR = 1.212, 95% CI = 1.024–1.434, *p* = 0.025), lesser degree of reduction in LA size after surgery (OR = 0.790, 95% CI = 0.960–0.980, *p* < 0.001), and large post-operative LA size (OR = 1.064, 95% CI = 1.045–1.083, *p* < 0.001) were risk factors for the occurrence of AF. Presence of MS rather than pure MR (OR = 1.767, 95% CI = 0.981–3.182, *p* = 0.058) was associated with the occurrence of AF with borderline significance (Table 3). Interestingly, none of the parameters reflecting pre-operative LA size was associated with post-operative AF. Pre and post-operative TR (OR = 1.864, 95% CI = 1.328–2.617, *p* < 0.001 & OR = 2.641, 95% CI = 1.922–3.630, *p* < 0.001) and postoperative higher PAP (OR = 1.067, 95% CI = 1.025–1.110, *p* = 0.001) were associated with the occurrence of AF. In multivariate analysis, post-operative LAVI was an independent predictor for the occurrence of AF (Table 4). The predictive ability of the LAVI was determined by the area under the curve of the receiver operating curve and post-operative LAVI > 39 ml/m^2^ (cut-off value) was associated with new onset permanent AF (sensitivity: 79%, AUC: 0.762, SE: 0.051, *p* < 0.001). The AF-free survival curves of patients with post-op LAVI < 39 ml/m2 or ≥39 ml/m2 are shown in Fig. 3 (*p* = 0.06).

**Table 1:** Baseline characteristics of the patients

Variables	NSR (*n =* 361)	AF (*n =* 81)	*p*
Age at surgery (years)	41 ± 13	41 ± 11	0.782
Male gender	151 (42%)	39 (49%)	0.264
Body surface area (m^2^)	1.65 ± 0.16	1.64 ± 0.16	0.967
Etiology			0.018
Rheumatic etiology	284 (79%)	73 (90%)	
Non-rheumatic valvular disease	77 (21%)	8 (10%)	
Diagnosis			0.065
Pure mitral regurgitation	205 (57%)	37 (46%)	
Presence of mitral stenosis	156 (43%)	44 (54%)	
Combined with other valve	180 (58%)	44 (70%)	0.091
Type of surgery			0.478
Mitral valve replacement	348 (96.4%)	79 (97.5%)	
Bioprosthesis	7 (2.0%)	2 (2.5%)	
Mechanical	341 (98.0%)	77 (97.5%)	
Mitral valve repair	13 (3.6%)	2 (2.5%)	
Annular size	28.4 ± 2.1	29.0 ± 2.6	0.070
Preoperative heart rate (bpm)	76 ± 19	75 ± 15	0.762
Preoperative blood pressure (mmHg)			
Systolic blood pressure	121 ± 17	123 ± 16	0.825
Diastolic blood pressure	75 ± 13	74 ± 10	0.623
Postoperative HR	73 ± 18	72 ± 14	0.854
Postoperative blood pressure			
Systolic blood pressure	119 ± 15	120 ± 15	0.718
Diastolic blood pressure	73 ± 11	74 ± 11	0.777

**Table 2:** Echocardiographic parameters before and after surgery

	NSR (*n =* 361)	AF (*n =* 81)	*p*
Before surgery			
LV end diastolic dimension (mm)	56.6 ± 10.8	55.0 ± 10.9	0.375
LV end systolic dimension (mm)	38.4 ± 9.2	38.7 ± 9.9	0.863
LV ejection fraction (%)	62.7 ± 10.0	58.9 ± 14.1	0.026
LA antero-posterior dimension (AP) (mm)	50.7 ± 8.7	53.1 ± 6.9	0.080
LA medio-lateral dimension (ML) (mm)	56.2 ± 10.6	58.6 ± 9.9	0.339
LA supero-inferior dimension (SI) (mm)	62.4 ± 9.7	60.4 ± 9.1	0.372
LA volume index (ml/m^2^)^a^	58.4 ± 24.9	62.9 ± 22.8	0.399
TR grade	0.4 ± 0.8	1.2 ± 1.1	0.005
Estimated PAP	39.2 ± 17.5	44.5 ± 18.4	0.252
After surgery			
LV end diastolic dimension (mm)	48.9 ± 5.3	50.1 ± 8.9	0.126
LV end systolic dimension (mm)	33.8 ± 5.8	35.9 ± 10.8	0.015
LV ejection fraction (%)	60.9 ± 9.5	57.2 ± 13.8	0.006
LA AP dimension (mm)	42.9 ± 6.0	50.7 ± 8.1	< .001
LA ML dimension (mm)	50.3 ± 6.8	56.3 ± 7.5	< .001
LA SI dimension (mm)	57.3 ± 27.5	61.8 ± 8.2	0.172
LA AP change (mm)	7.7 ± 8.4	4.1 ± 7.9	0.011
LA ML change (mm)	6.7 ± 10.4	3.4 ± 10.1	0.197
LA SI change (mm)	7.4 ± 9.0	1.3 ± 8.3	0.005
LA volume index (ml/m^2^)	37.9 ± 12.6	52.1 ± 15.6	< .001
LA volume change (ml/m^2^)	20.5 ± 21.3	10.8 ± 23.4	0.041
LA volume change %	28.1 ± 27.9	9.5 ± 36.8	< .001
MDPG of the MV (mmHg)	3.5 ± 1.4	3.9 ± 1.5	0.023
Residual mitral regurgitation			0.337
No	352 (97.5%)	77 (95.1%)	
Trivial	8 (2.2%)	3 (3.7%)	
More than grade I	1 (0.3%)	1 (1.2%)	
TR grade	0.3 ± 0.6	1.0 ± 1.0	< 0.001
Estimated PAP	26.4 ± 6.6	29.8 ± 7.1	0.001
TAP or TVR	33 (7.5%)	6 (1.4%)	0.828

^a^LA volume index was available only in 200 patients (176 of NSR, 24 of AF); *NSR* normal sinus rhythm, *AF* atrial fibrillation, *LV* left ventricular, *LA* left atrial, *MDPG* mean diastolic pressure gradient, *TR* tricuspid regurgitation, *PAP* pulmonary artery pressure, *TAP* tricuspid valve repair with an annuloplasty ring, *TVR* tricuspid valve replacement

**Table 3:** Factors determining the occurrence of atrial fibrillation after surgery (univariate analysis)

Variables	Odds ratio	95% confidence interval	*p*
Rheumatic VHD	2.474	1.143–5.355	0.021
Presence of MS	1.767	0.981–3.182	0.058
Preoperative LV EF	0.971	0.949–0.992	0.028
Postoperative LV EF	0.970	0.949–0.992	0.007
Δ LAVI	0.960	0.940–0.990	0.007
% Δ LAVI	0.790	0.960–0.980	< .001
Postoperative LAVI	1.064	1.045–1.083	< .001
Postoperative MDPG	1.212	1.024–1.434	0.025
Preoperative TR	1.864	1.328–2.617	< 0.001
Postoperative TR	2.641	1.922–3.630	< 0.001
Postoperative PAP	1.067	1.025–1.110	0.001

*VHD* valvular heart disease, *MS* mitral stenosis, *LV* Left ventricular, *EF* ejection fraction, *Δ* change, *LAVI* left atrial volume index, *MDPG* mean diastolic pressure gradient, *TR* tricuspid valve regurgitation, *PAP* pulmonary artery pressrue

**Table 4:** Factors determining the occurrence of atrial fibrillation after surgery (multivariate analysis)

Variables	Odds ratio	95% confidence interval	*p*
Rheumatic VHD	4.683	0.716–30.460	0.107
Presence of MS	3.534	0.884–14.125	0.074
Preoperative LV EF	0.997	0.953–1.043	0.815
Postoperative LV EF	1.004	0.957–1.054	0.863
% Δ LAVI	0.993	0.976–1.010	0.409
Postoperative LAVI	1.098	1.047–1.153	< .001
Postoperative MDPG of MV	0.902	0.557–1.459	0.673
Preoperative TR	0.686	0.181–2.596	0.579
Postoperative TR	2.274	0.896–5.773	0.084
Postoperative PAP	0.930	0.788–1.097	0.389

*VHD* valvular heart disease, *MS* mitral stenosis, *LV* Left ventricular, *EF* ejection fraction, *Δ* change, *LAVI* left atrial volume index, *MDPG* mean diastolic pressure gradient, *MV* mitral valve, *TR* tricuspid valve regurgitation, *PAP* pulmonary artery pressrue

## Discussion

In the present study, the prevalence and predictors of new onset permanent AF after MV surgery were investigated. The results showed that new onset permanent AF is not uncommon, occurring in about 20% of patients even after successful MV surgery and pre-operative sinus rhythm. Although several parameters, such as rheumatic etiology of MV, presence of mitral stenosis (MS), lower LV EF, higher post-operative mean diastolic pressure gradient across mitral prosthesis, and lesser degree of reverse LA remodeling after surgery, were shown to be associated with the occurrence of post-operative AF, post-operative LAVI was found to be an independent predictor for the occurrence of AF in multivariate analysis. Interestingly, none of the parameters reflecting pre-operative LA size was associated with post-operative AF. These findings underscore the importance of post-operative echocardiographic assessment before dismissal of the evaluation of LV function and LA size even in patients who have undergone successful MV surgery and pre-operative sinus rhythm.

### Factors associated with new onset permanent AF

#### Reverse LA remodeling and post-operative AF

The degree of LA reverse remodeling was different in the two groups and showed that the reduction of LAVI and decrease in percentage of LAVI were smaller in the post-op AF group. Accordingly, post-operative LA size was significantly larger in the post-op AF group. In a previous study, post-operative LAVI ≥60 ml/m^2^ was shown to be associated with adverse clinical outcomes in patients with organic mitral regurgitation (MR) [19]. In our study, 9 (47%) of 19 patients who had post-operative LAVI ≥60 ml/m^2^ developed new onset permanent AF, whereas 15 (8%) of 181 patients who had post-operative LAVI < 60 ml/m2 had new onset AF (*p* < 0.001). These findings are consistent with previous research [20]. However, in our study, post-operative LAVI ≥39 ml/m^2^ was selected as a cut-off value for new onset AF. It had a sensitivity of 79% as well as the largest AUC (0.762). The smaller cut-off value of LAVI in our study was probably due to the inclusion of the patients with MS in our study. Regarding the direction of LA reverse remodeling, the change in SI dimension was the most prominent when compared with that of the AP and ML dimensions. Therefore, assessing the change in LA size only in the AP dimension may not accurately reflect the change in LA size. Thus, the degree of LA reverse remodeling could be underestimated.

#### Impact of LV systolic function on post-operative AF

In our study, lower pre- and post-operative LV EF was also associated with new onset AF. Despite similar pre-operative LAVI, lesser degrees of reduction in LAVI and decreases in percentage of LAVI occurred in patients with LV EF < 60%. LV systolic dysfunction usually accompanies LV diastolic dysfunction and elevated LV filling pressures. Despite similar LA size, the presence of LV systolic dysfunction and concomitant LV diastolic dysfunction with elevated LV filling pressure might interfere with LA reverse remodeling.

#### Pre-operative etiology of MV pathology

New onset permanent AF occurred more frequently in patients with rheumatic etiology and presence of MS rather than pure MR. Because LA kinetic energy is different in MS and MR, a long-standing pressure overload of the LA in MS might be associated with higher LA kinetic energy than volume overload in MR. Therefore, increased LA work in MS may result in further LA fatigue and failure over time, which may disturb LA reverse remodeling [2, 21].

### Role of post-operative echocardiography before dismissal

Although successful intervention was performed on diseased MV, AF can occur in patients with post-operative LAVI ≥39 ml/m^2^ according to the results from our study. Therefore, it is imperative to not only focus on the successful results of valve surgery but also perform post-operative echocardiography to evaluate post-operative LAVI to predict the occurrence of AF.

Echocardiography has a different role in the evaluation of valvular heart disease (VHD) at different stages before and after surgery. Pre-operative TTE should provide an accurate diagnosis to determine the possible cause of valvular diseases [22, 23]. In addition, quantitative echocardiographic evaluation of LV size and function is a key factor in clinical decision making in adults with VHD [24–27]. Other key echocardiographic data includes LV diastolic function, LA enlargement, and the presence of intra-cardiac thrombus, pulmonary artery pressures, and so on [28]. Despite the important information obtained from pre-operative echocardiography, none of the pre-operative echocardiographic parameters provides predictive information regarding post-operative permanent AF in this study. Intra-operatively, transesophageal echocardiography (TEE) provides a roadmap for the surgeons regarding the location and severity of MV pathologic lesions, enhancing the ability to detect unexpected associated lesions. In addition, intra-operative TEE is used to confirm results of surgical procedures on the MV, which can result in improved surgical outcomes [28, 29]. Therefore, the American College of Cardiology and the American Heart Association have established guidelines for the management of patients with VHD, which state that the use of intra-operative TEE in MV repair is a class I indication [30].

Although the importance of pre-operative and intra-operative echocardiographic evaluations of MV disease has been well recognized, the role of post-operative pre-discharge TTE has been overlooked in patients undergoing MV surgery. Unlike pre-operative echocardiographic parameters, post-operative LAVI measured before dismissal was able to predict the occurrence of post-operative permanent AF in patients undergoing MV surgery. Based on these results, the importance of post-operative echocardiographic assessment is emphasized not only for assessing the results of MV surgery but also for evaluating cardiac chamber size and function, particularly LA volume.

### Limitations

The current study has several limitations. First, it was a retrospective study, so the data collection was done by reviewing medical charts and recorded echocardiographic data. Therefore, the measurement of LAVI was available in only 200 of 442 patients, and only echocardiographic report was available in the remainder. And, the majority of the study patients had undergone MVR rather than MV repair. Anticoagulation is one of the most important treatments for AF. However, all patients should have life-long anticoagulation therapy for the implanted valve, except for MV reconstructions and bioprosthetic valve implantation. Therefore, our results cannot apply to patients who have undergone MV repair.

## Conclusions

Newly developed postoperative permanent AF is not uncommon, occurring in 18% of patients who have undergone successful MV surgery despite pre-operative sinus rhythm. Rheumatic etiology, the presence of MS, lower LV EF, lesser degree of LA reverse remodeling, and larger post-operative LAVI were associated with new onset permanent AF. Post-operative LAVI > 39 ml/m2 was an independent predictor for the occurrence of AF.

## Abbreviations

AF: atrial fibrillation
AP: anteroposterior
CI: confidence interval
ECG: electrocardiogram
EF: ejection fraction
LA: left atrial
LAVI: left atrial volume index
LV: left ventricular
MDPG: mean diastolic pressure gradient
ML: mediolateral
MR: mitral regurgitation
MS: mitral stenosis
MV: mitral valve
NSR: normal sinus rhythm
OR: odds ratio
ROC: receiver-operating-characteristic
SD: standard deviation
SI: superior/inferior
TEE: transesophageal echocardiography
TTE: transthoracic echocardiography
VHD: valvular heart disease
